# A patient-centered view of symptoms, functional impact, and priorities in post-COVID-19 syndrome: cross-sectional results from the Québec Action Post-COVID cohort

**DOI:** 10.1186/s12879-025-10757-1

**Published:** 2025-04-02

**Authors:** Nancy E. Mayo, Marie-Josée Brouillette, Emilia Liana Falcone, Lesley K. Fellows

**Affiliations:** 1 Dept of Medicine, and School of Physical and Occupational Therapy, Faculty of Medicine and Health Sciences, McGill University, Montreal, QC Canada; 2 Center for Outcomes Research and Evaluation (CORE), MUHC-Research Institute, 5252 de Maisonneuve, Office 2B:43, Montreal, QC H4A 3S5 Canada; 3 Dept of Psychiatry, McGill University, Montreal, QC Canada; 4 Center for Inflammation, Immunity and Infectious Diseases, Montreal Clinical Research Institute (IRCM), Montreal, QC Canada; 5 Dept. of Medicine, Université de Montréal, Montreal, QC Canada; 6 Faculty of Medicine and Health Sciences, McGill University, Montreal, QC Canada; 7 Dept of Neurology & Neurosurgery, Montreal Neurological Institute, McGill University, Montreal, QC Canada

**Keywords:** COVID-19, Post-COVID syndrome, Symptoms, Function, Quality of life, Fatigue, Post-exertional malaise, Brain fog, Cognition, Health services, Rehabilitation

## Abstract

**Background:**

Health services planning and mechanism-focused research would benefit from a clearer picture of symptoms, impact, and personal priorities in post-COVID-19 syndrome (PCS). This study aimed to provide estimates of the symptom, function, and quality of life (QOL) impact of PCS.

**Methods:**

People living in Quebec, aged ≥ 18, were eligible for the Québec Action for/pour le Post-COVID (QAPC) study if they had symptoms lasting more than 4 weeks post-acute SARS-CoV-2 infection, with or without a positive COVID-19 test. Recruitment was through conventional and social media between September 2022 and December, 31, 2023. Standardized and individualized questionnaires, in French or English, were accessed through an online portal. We report cross-sectional results from the baseline visit.

**Results:**

Individuals (*n*=535) spontaneously reported symptoms attributable to an average of 4.5 organ systems. Fatigue was most frequent. Effects on function and quality of life were moderate to severe and had already persisted for a year or more in the majority. Personal intervention priorities included fatigue and post-exercise malaise (PEM), cognitive symptoms, shortness of breath, and impaired taste and smell. Except for PEM, women and men did not differ importantly on measures of PCS impact, while older age was associated with lower impact.

**Conclusion:**

Symptom clusters defined a range of severity, with fatigue a pervasive symptom at all levels of severity. Participants in this study are likely to be representative of those seeking health care for post-COVID-19 symptoms in Canada and the results can inform next steps for clinical, research, and health services planning.

## Background

As of January 2023, it was estimated that 4.5 million Canadians have been infected with SARS-CoV-2, including 1.3 million in Quebec. [1, 2] It became evident early in the pandemic that symptoms could persist or arise after the acute infection. In Quebec, the Institut National d’Excellence en Santé et Services Sociaux recognizes post-COVID-19 syndrome (PCS) when symptoms last more than 12 weeks [3], the same time frame as the WHO [4]. Prevalence estimates from population-based studies around the world range from 3 to 70% [5–14] depending on the study sample, timeframe, and methodological rigour. Statistics Canada estimates prevalence at 14.8%, some 1.4 million people [14]. Symptoms also vary in nature and frequency across studies, with over 200 symptoms reported. [15] Few studies have reported on function, health-related quality of life (HRQL) or quality of life (QOL), but available evidence suggests substantial negative impacts. A recent structured review [16] found four studies reporting on the impact of PCS on quality-adjusted life-years based on the EQ-5D, finding a health reduction equivalent to losing 3 to 4 years of a hypothetical 10-year lifespan, values that are in the same range as chronic stroke, multiple sclerosis, and diabetes [17–19].

Facing a new and poorly understood health condition, the Quebec Action for Post-COVID (QAPC) study aimed to contribute a patient-centered understanding of symptom patterns, impact, and intervention priorities in a self-identified Quebec sample. The objectives of this initial report were to estimate the prevalence and severity of the health effects and life impact of PCS and the extent to which these differed by age and sex. In addition, while multiple symptoms can potentially affect those with the condition, we aimed to identify which of these symptoms are most bothersome for those with the condition, information that could guide the development of services to address these areas of priorities. The study used well-validated patient-reported outcome measures (PROMs), including the Patient Generated Index (PGI), an individualized measure suited to eliciting the most frequent and most bothersome symptoms) [20]. This fully virtual bilingual study further aimed to empower participants by providing information regarding their own health profiles and access to self-management resources.

## Methods

A cross-sectional analysis of people recruited into QAPC from September 23, 2022, to December 31, 2023, was carried out involving people from Quebec who self-identified as having symptoms of the post-COVID-19 syndrome. The sample was assembled from multiple sources: most participants were reached through French- and English-language media (radio) and social media, with some contacted via email outreach to a waitlist for a post-COVID research clinic. Residents of Quebec age 18 and over were eligible if they currently had symptoms occurring 4 or more weeks post onset of symptoms of the COVID-19 infection, with or without a positive test.

### Procedures

The project (2022–8066) was approved by the Research Ethics Board of the McGill University Health Centre. People interested in participating were directed to the QAPC website to register. The study coordinator recorded their contact information, generated a study identification number and invited them into the study. Upon invitation, they were directed to an online web-portal “Research Electronic Data Capture” (REDCap) to enter their unique identification number, allowing them entry into the data capture platform. Following an e-consent process, they recorded their health outcomes information. All were asked to consent to open data sharing for secondary analyses and to be re-contacted for additional studies.

Recruited participants also were provided a password to access online self-management resources addressing breathing difficulties, cognitive symptoms, fatigue, and increasing physical activity. To manage mental health symptoms, participants were directed to BounceBack®, a free program offered by the Canadian Mental Health Association to help manage low mood, mild to moderate depression, anxiety, stress or worry [21].

### Measurement framework

The Wilson-Cleary model guided the health outcomes assessment [22]. A lightweight yet comprehensive approach used patient-centered assessment tools to characterize symptoms and their functional and quality of life impact at recruitment and over time.

As little was known at this study’s outset about the health effects beyond a symptom inventory [23], we adopted an individualized approach to outcome measurement. The Patient Generated Index (PGI) [20] asks people to nominate areas of their life affected by a health situation, here the sequelae of COVID. Each area is then rated on severity, from 0, “not at all”, to 10, “worst imaginable severity”. The person is then asked to consider areas where they most desire improvement and distribute a theoretical 10 tokens across the nominated areas. A total score is generated by multiplying the severity rating by the number of priority tokens allocated and summed. This process avoids the over-reporting that can occur when people are asked to choose from a list of symptoms, rather than spontaneously declaring them [24], and provides information about priorities in the person’s own words.

The only measure that has been recommended to date for assessing the health impact of post-COVID is the SF-36 [25]. We used it in its publicly available form (RAND-36) [26]. We also used an internationally recognized health utility measure, the EQ-5D-5L [27, 28], which queries 5 functions: walking, usual activities, self-care, pain/discomfort, and anxiety/depression. These were supplemented with a series of visual analogue scales to cover the additional areas of fatigue, sleep, distress, shortness of breath (SOB), health rating, and overall quality of life [29, 30]. Two additional single questions queried fatigue impact [31] and minutes of exercise over a week. Questionnaires on post-exertional malaise (PEM) [32], post-traumatic stress disorder (PTSD) [33] and cognitive concerns [34] were also administered.

People were asked to do a home smell test, the Yale Jiffy Test, using peanut butter, an olfactory stimulus, and vinegar, a trigeminal stimulus. [35] Strength of smell was measured on a 7-point ordinal scale: no sensation; barely detectable; weak; moderate, strong; very strong; and strongest imaginable. The first three categories indicated low olfactory detection.

Demographic information, co-morbidity, and COVID experience and vaccine history were also collected. Assessment tools had strong evidence for validity with robust measurement properties, were available in English and French, and in most cases had existing Canadian or Quebec norms [36–38] or comparative values [34] aiding interpretation. Each participant’s information was summarized, interpreted, and shared with them in the form of a personal dashboard, similar to one we had previously designed and tested for people living with HIV [39].

### Data analysis

Descriptive statistics were calculated for each variable for the full cohort and after recruitment of approximately 100, 200, 300 and 400 participants. Values on study measures did not vary across these waves suggesting that early and late responders did not differ on severity. Two people had missing data on language and on hospitalization. Where relevant, the effect of sex and age were estimated using linear, quantile (median), ordinal, or logistic regression depending on the distribution of the variable under study. When there was a sex effect, data were presented separately for men and women. As the sample size was large enough to show statistical significance for small differences, a sex effect was considered important when confidence interval around the estimated effect excluded the null value and the difference in prevalence between men and women was greater than 10% [40]; for continuous variables an important difference was considered to be ½ standard deviation or greater. [41] Text threads were analysed using natural language processing (i.e., stemming and lemmatization).

Cluster analysis (k-means) was used to identify the extent to which the functional consequences of fatigue, cognition, sleep, distress, physical function, and shortness of breath clustered in individuals. For this analysis, all variables were converted to a score out of 100, with 100 being the worst level. The elbow plot was used to identify the optimal number of clusters. The association between symptom burden of PCS and health and quality of life outcomes and work status, were estimated using linear regression or logistic regression by regressing these outcomes on cluster membership, adjusted for age and sex. The association of cluster with smell was similarly estimated. The quantitative analyses were conducted using SAS V9.4.

## Results

A total of 555 people registered for the study, but data were not available on all variables for all people. Table 1 presents personal and COVID-related characteristics of the sample. Information on age and sex was available for 535 people, mean age 48.8 years; women predominated (76.1%). The hospitalization rate of 6% was similar to the rate in the Canadian population. Older people were more likely to be hospitalized that younger people and men spent more days in hospital than women. The vaccination rate of the sample was greater than the Quebec population. The date of the last COVID event was reported for 524 people, and the average time from last event was 324 days. Reported substance use ranged from ≈5% for smoking and cannabis use to 50.7% for monthly or more alcohol consumption and 16.5% reported increased consumption. Almost 80% of the sample were working prior to COVID and over 68.8% of those reported changing their working hours with PCS; 42% reported being on sick leave. Even with PCS, the sample reported carrying on with usual roles and responsibilities, with women having more household responsibilities than men. Almost half had money challenges.

**Table 1:** Characteristics of the Sample (*n* = 535)

Sociodemographic [Norm]	N or Mean	% or SD	Model
Sex: Men/Women/Other	121/407/7	22.5%/76.1%/1.3%	
Age	48.8	12.2 (18–83)	
Men (*n* = 121)	51.1	13.2	
Women (*n* = 407)	48.4	11.6	
Education			
High school	47	8.8	
College or technical	159	29.7	
Bachelors	176	32.9	
Post-graduate / professional	153	28.6	
Language: English/French	184/371	33.1%/66.9%	
Caucasian race	452	84.5%	
Positive test for COVID	486	92.1%	
Hospitalized [5.8%]^a^	29	6.0%	Logistic
Men	12	10.9%	
Women	16	4.3%	
Days in hospital: M/W	14.7/ 7.1	ne	Quantile
Range	1–58		
1 day	11	37.3%	
2–6 days	8	28.6%	
7–14 days	5	17.8%	
> 14 days	5	17.8%	
ICU / Intubated	12	42.9%	
COVID more than once	152	28.8%	Logistic
Time from last + COVID test (days)	524	324.4 (251.0) [129—434]	Quantile
Reported number of vaccines			
0	12	2.3%	
1 + [80%]	516	97.7%	Logistic (1 +)
2 +	502	95.1%	
3	390	73.9%	
4^a^	207	39.2%	
5	37	7.0%	
Missing	27		
Current smoker	26	4.9%	Logistic
Cannabis user	30	5.6%	Logistic
Alcohol consumer (> monthly)	271	50.7%	Logistic
Any increase consumption	88	16.5%	Logistic
Working prior to COVID^a^	426	79.6%	Logistic
Hours have changed	293	68.8%	Logistic
On paid / unpaid sick leave	179	42.0%	Logistic
Roles and responsibilities outside work			
Pre-school children^b^	49	8.8	Logistic
School-age children^b^ M/W	32/153	26.5%/38.15	Logistic
Caring for pets^a^ M/W	48/233	39.7/57.3	Logistic
Shopping M/W	85/334	70.3%/82.1%	Logistic
Meal preparation^b^ M/W	75/345	62.0%/84.8%	Logistic
Household management M/W	88/350	72.7%/86.0%	Logistic
Others	117	21.1%	
Money to meet needs: not completely^a^	265	49.6%	Logistic
Worry about money^a^	308	57.7%	Logistic

^a^age effect: older adults more likely to be hospitalized and receive 4 or more vaccines

^b^age effect: younger more work and other responsibilities

Canadian data on hospitalization and vaccination rates from Canadian Government [42]

**Fig. 1:**
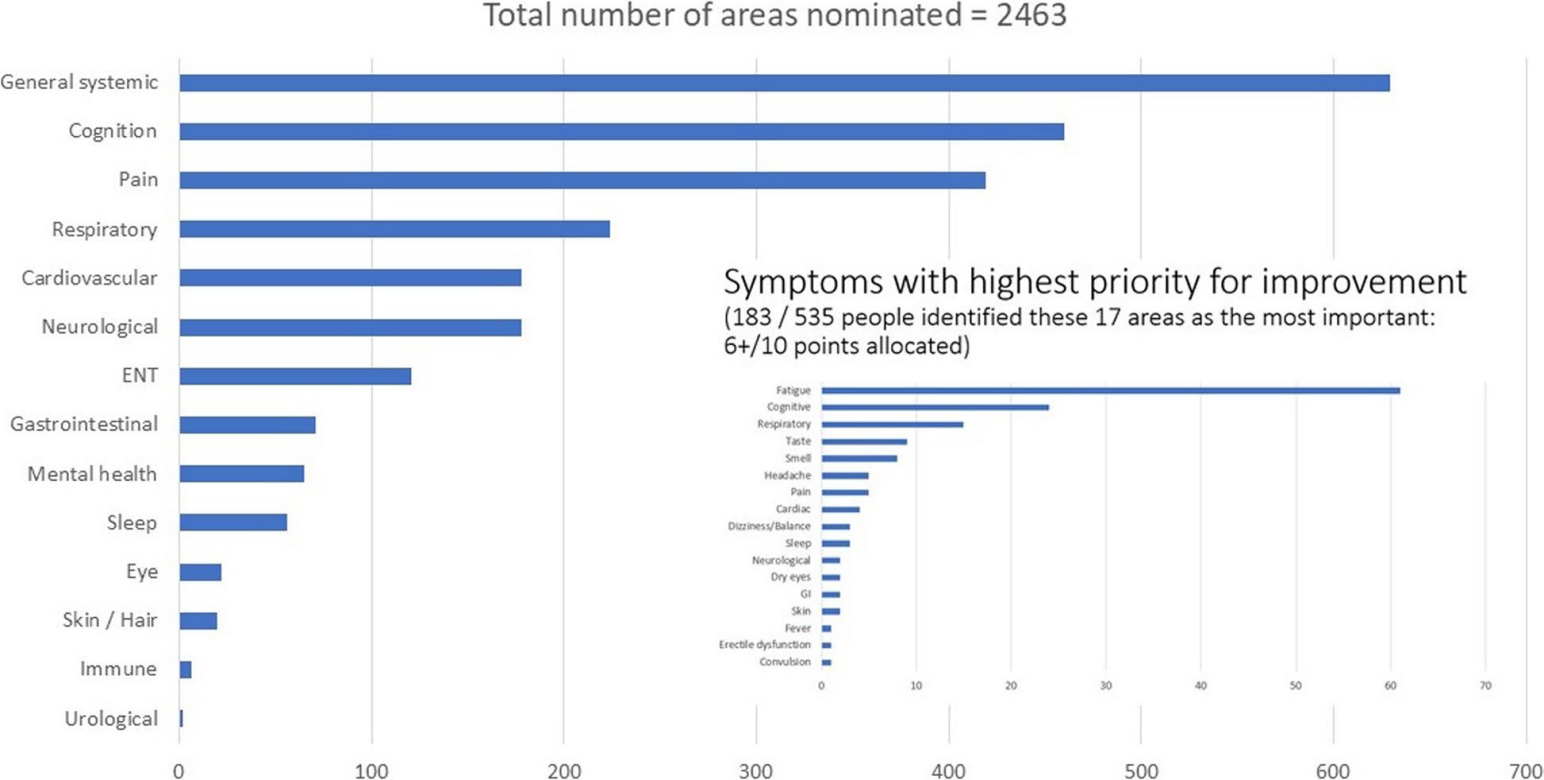
Most frequently nominated symptoms according to organ system and symptoms with highest priority for improvement

Figure 1 shows the number of areas nominated by people with PCS according to organ system. General systemic symptoms (which includes fatigue) were the most frequently nominated followed by symptoms related to cognition, pain, and the respiratory, cardiovascular and neurological systems. The symptoms with the highest priority for improvement were fatigue, cognition, respiration, taste, and smell.

Table 2 presents values on measures of symptoms. The fatigue construct was represented in four different ways. Fatigue severity was high, averaging 64.9 / 100 with 100 as the worst fatigue. Among women, 86% reported PEM compared with 74% of men. Over 1/3 identified that their fatigue required resting most of the day. One quarter of the sample reported symptoms indicative of PTSD and over 28% reported severe SOB affecting daily activities. Approximately 15% showed lowered olfaction for smelling peanut butter and vinegar. Over 1/3 were prescribed medications for the health effects of PCS. Loneliness was reported by over 60% of the sample compared with 10.4% of the general Canadian population of similar age; 21% reported irritability.

**Table 2:** Symptom, function, health, and QOL impact of PCS

Symptom [Norm]	N or Mean [Median]	% or SD	Model
Fatigue			
Severity^a^ (VAS 0 −100 worst)	64.9 [70]	24.6	Quantile
Post-exertional malaise (PEM)^a^			Logistic
Men	90	74.4%	
Women	350	86.0%	
Need to rest during the day^a^			
No	51	9.3	Referent
1 + times during the day	297	54.1	Ordinal
Most of the day	201	36.6	Ordinal
Sleep^a^ (VAS 0 −100 worst)	49.8 [51.0]	29.7	Quantile
Distress			
Severity (VAS 0 – 100 worst)			Quantile
PTSD (≥ 3/5 symptoms)	131	25.1	Logistic
Shortness of breath (SOB)			
Severity (VAS 0 – 100 worst)	36.1 [31]	28.8	Quantile
Breathlessness			
SOB walking 100 m on level ground	89	16.2%	Logistic
SOB dressing / can’t leave house	32	5.8%	Logistic
Reduced Olfaction			
Peanut butter	78	14.8%	
Vinegar	77	14.8%	
Prescribed medication for PCS symptoms	200	37.4	Logistic
Lonely sometimes or often [10.4%]	325	60.1	Logistic
Irritable often to always	112	21.0	Logistic
Cognition:C3Q (0–100 best) [81]	43.0 [38.9]	26.0	Linear
Most prevalent symptoms of concentration loss			
Too many thoughts in my head	327	60.1	
Can’t pay attention to two things at a time	315	57.9	
Can’t do complex tasks	291	53.5	
Can’t reading more than a few pages	275	50.6	
Can’t pay attention to verbal instructions	254	46.7	
Most prevalent memory symptoms			
Forgot what I was about to do	316	58.1	
Forgot if what I have just read	248	45.6	
Forgot tasks or activities I need to do	232	42.7	
Forgot what I have already done something	210	38.6	
Forgot what I was supposed to buy at the store	209	38.4	

Norms for C3Q are from Askari et al. [34]

Population rate of loneliness are from Statistics Canada [35]

^a^age effect: older adults had lower symptom burden

Cognitive function was poor with people reporting cognitive challenges in everyday life activities. On a self-report measure of cognitive concerns (C3Q), the average value out of 100 was 43 compared with 81 for a comparative sample. The most prevalent challenges were related to concentration with over 50% reporting challenges.

Table 3 shows the impact of PCS on health and QOL outcomes. The values on the subscales (higher is better) of the RAND-36 were substantially lower than normative values. There were no important differences in the RAND-36 subscales by sex except for Role Emotional with women having lower values. Values on the PGI (mean: 26.6) are lower than for other methods of measuring QOL (means ≈38) as this format is based on people nominating only negative aspects of their life.

**Table 3:** Health-related quality of life outcomes

HRQL Measures (0–100 best)	Norm (45–54 years)	Mean (SD)	Median [1QR]	Model
**RAND-36**				
Physical Function	86.6	52.3 (27.9)	50 [30–75]	Quantile
Role Physical	82.0	11.5 (26.0)	0 [0–0]	
> 0 vs 0		21.2%	*n* = 431/116	Logistic
Bodily Pain	72.9	46.7 (25.1)	41 [31–62]	Quantile
General Health^a^	77.2	41.9 (23.0)	40 [25–60]	Quantile
Vitality^a^	63.3	23.4 (19.2)	20 [10–35]	Quantile
Social Function	84.3	35.4 (27.0)	25 [12.5–50]	Quantile
Role Emotional	84.2	38.5 (42.9)	33.3 [0–100]	Ordinal
Men (% ≤ median)			71.9%	
Women (% ≤ median)			60.9%	
Mental Health^a^	75.6	54.3 (20.4)	56 [40–68]	Linear
**EQ-5D Index**^a^ (0 – 1 best)	0.80	0.63 (0.22)	0.68 [0.51 – 0.81]	Linear
**General health VAS**		38.5 (22.6)	35.0 [22.0 – 50.0]	Linear
**QOL VAS**		38.1 (24.2)	35.0 [20.0 – 50.0]	Linear
**PGI**		26.6 (14.6)	25.0 [17.0 –35.0]	Linear

^a^age effect, older is better

Norms for RAND-36 are from Hopman et al. [36]; Norms for EQ-5D Utility are from Poder et al. [37]

**Fig. 2:**
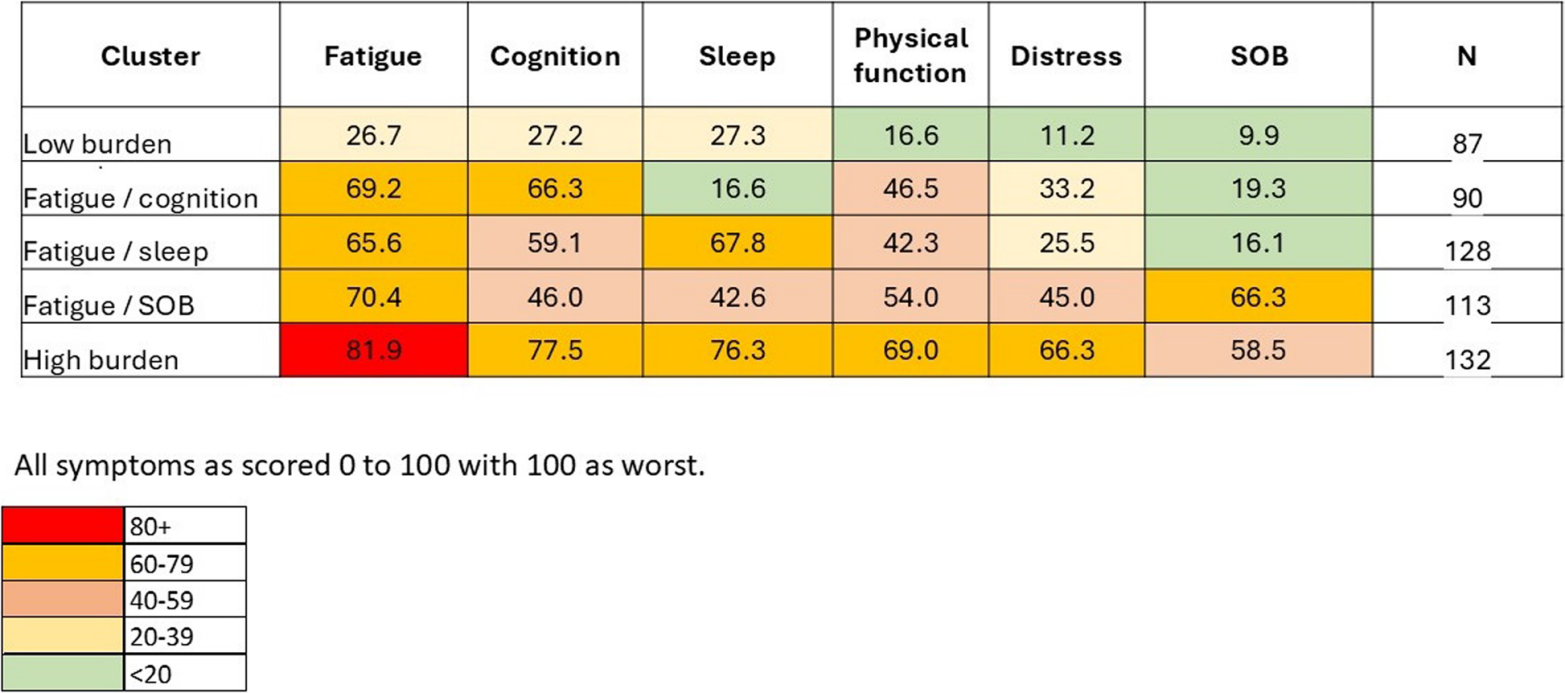
Symptom clusters

Figure 2 shows the results of the cluster analysis. Five clusters were identified mainly distinguished by the degree of symptom burden with fatigue severity as a defining variable.

**Fig. 3:**
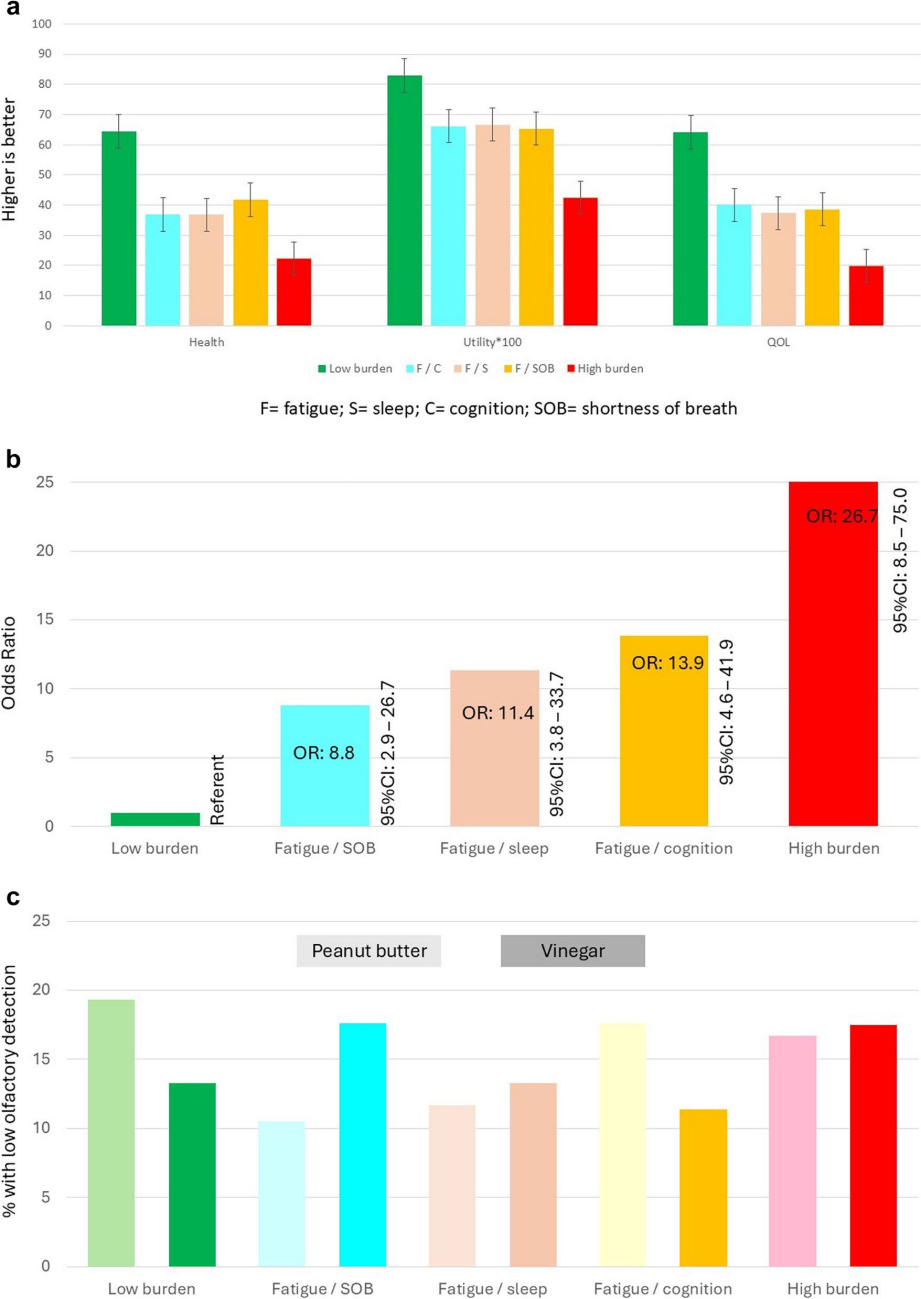
**a** Association of Symptom Cluster with Health and QOL Outcomes. **b** Association of Symptom Cluster with Sick Leave from Work. **c** Association of Symptom Cluster with Olfaction

**Fig. 4:**
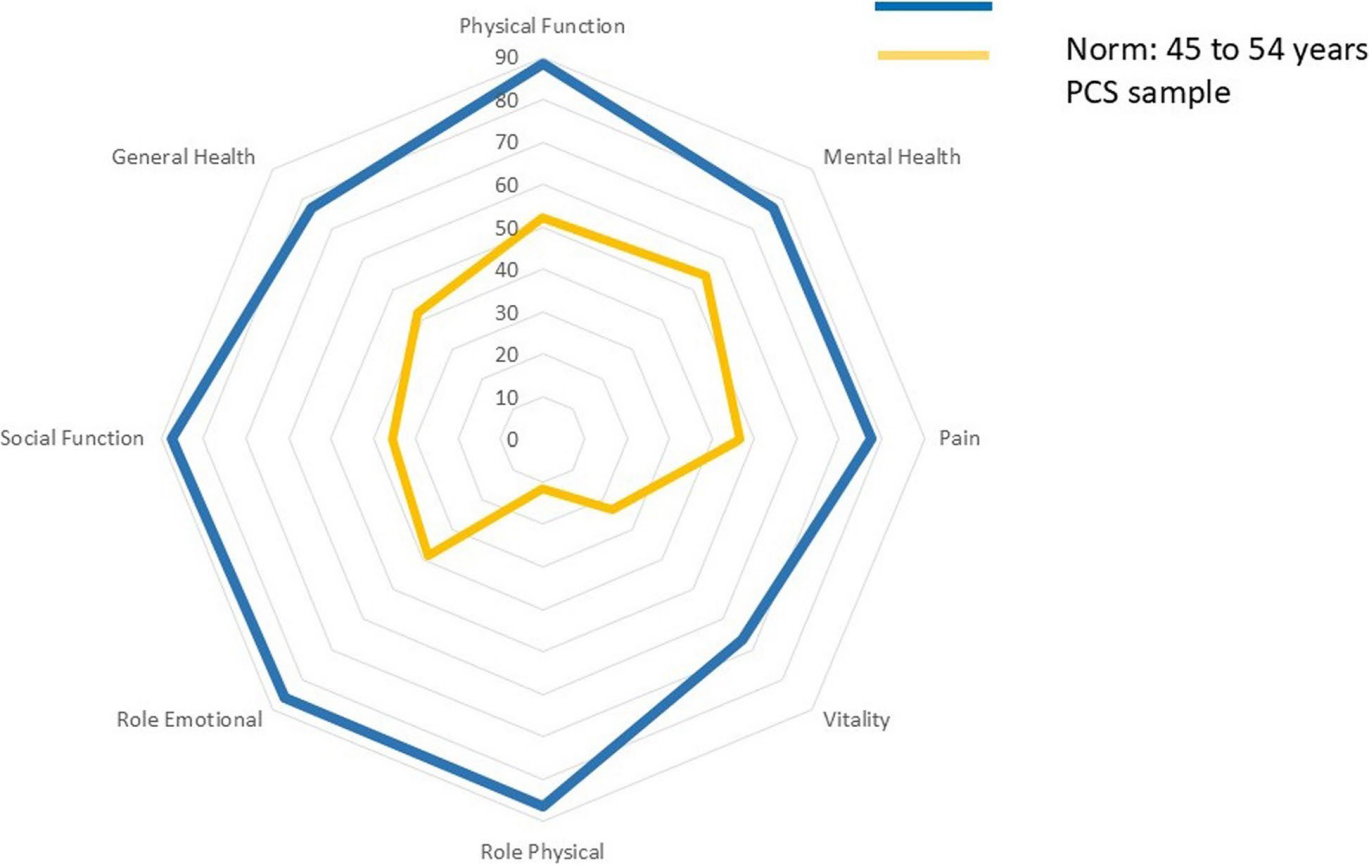
Values on RAND-36 Subscales for People with PCS and Normative Values for people 45–54 years

The effects of cluster membership on health and QOL outcome, estimated using linear regression with adjustment for age and sex, are shown in Fig. 3a. There was a significant cluster effect for all outcomes. Cluster membership explained over 30% of the variability in these health outcomes and there was an age effect only for the EQ-5D index value with older age associated with higher health utility. Figure 3b shows that, in comparison to people in the lowest symptom burden cluster, there was an increasing proportion of people on unpaid sick leave, according to cluster membership. Figure 3c shows that there was no association of symptom cluster to olfactory sensation loss (Chi-square 25.8, 24df; *p* = 0.3655). Figure 4 illustrates the impact of PCS on HRQL as measured by the RAND-36.

## Discussion

This study of adults self-identifying with PCS in Quebec, Canada found long-lasting, widespread, and severe effects across the full range of health outcomes (symptoms, function, health perception, and QOL). The symptom of most concern to this sample was fatigue, with over 80% reporting PEM and over 90% needing to rest during the day. Cognitive symptoms were also very prevalent with people scoring 40 (out of 100) points lower than expected on a measure of self-reported cognitive concerns. Concentration difficulties were the most common cognitive issue. Respiratory symptoms were also frequent. Symptoms had substantial functional impact, with close to half the sample on sick leave. Health-related quality of life as assessed with the EQ-5D index values for the QAPC sample was 0.63, comparable to values reported for Canadians with stroke, multiple sclerosis, or advanced cancer [17, 18] and far below the normative value for Quebec (0.80). [38].

A similar profile of PCS burden has been reported by others [15, 23, 43–48] although it is difficult to compare studies owing to the differences in how samples were assembled. PCS is a self-reported syndrome, and hence how people are recruited into studies will affect estimates of symptom burden. The QAPC sample was predominately women (76%), as in other studies with self-identified PCS samples [42]. In contrast, in the UK census study that is the best available population-level data on self-reported PCS to date [6], the women to men ratio was ≈3:2. However, we found that women and men were similar on the outcomes we assessed. We observed effects of age on some variables. Paradoxically, while older age was associated with higher likelihood of hospitalization during acute infection, it was associated with lower PCS symptom burden and better QOL.

We assessed symptom clustering, seeking evidence for sub-syndromes that might have different etiologies or contributors. The observed pattern is consistent with a range of severity rather than sub-syndromes, with fatigue an early feature in even the mildest stages, and additional symptoms accruing with increasing severity.

Cluster profiles were associated with health outcomes such as HRQL and QOL, with the least and most burdensome clusters having the highest and lowest values on these health outcomes, respectively (see Fig. 3a). There was also a dose–response relationship with cluster burden and proportion of people on sick leave (see Fig. 3b). While change in olfaction was rated as one of the most burdensome symptoms (see Fig. 1), impaired olfaction as assessed by perceived intensity of the smell of peanut butter was not associated with symptom cluster (see Fig. 3c). The proportion of people reporting weak olfaction to peanut butter and vinegar was nearly identical at 14.8% (see Table 1). Chudziz et al. reported that 4.4% of people still reported loss of smell 3 months after COVID infection [49].

A recent systematic review of 151 studies from 32 countries with a total of 1,285,407 participants who would meet criteria for PCS [12] reported that the proportion of people with at least one symptom decreased over time from 56.0% (at 1–3 months) to 37.8% (at 6–12 months). This indicates that over a third of people reporting PCS can be symptomatic for a year or more. While the cross-sectional observations here do not address the evolution of PCS, participants had symptoms for nearly a year at study entry, on average. On-going longitudinal follow-up will shed light on the evolution and day-to-day variability in this seemingly chronic phase of COVID-19.

This study is affected by the same limitations as much of the existing literature on PCS, as there is no base population to sample from and people entering the study were likely those with the most severe and persistent symptoms. Further, while over 90% of the QAPC sample reported a positive COVID test, infection status was not independently verified. There was widespread availability of PCR testing in the early waves of the pandemic in Quebec, and home tests were provided free of charge in pharmacies thereafter. Study participants likely had SARS-CoV-2 infection with persistent disabling chronic symptoms and were people most likely to seek help. The sample was infected and recruited during the pre-Omicron period [50] when symptoms were most severe [51]. This could explain the very high burden of PCS symptoms, particularly PEM.

While the findings from this self-identified sample do not provide information on prevalence, they can inform healthcare service planning: People identified relief from fatigue, pain, and from cognitive, respiratory, and cardiac symptoms as their top priorities for treatment. This information could help prioritize current management recommendations which are based on reported frequency and expert opinion [52] Rehabilitation approaches may be particularly helpful, given the nature, functional impact, and chronicity of the most bothersome symptoms. Most people had multiple symptoms, indicating that a multidisciplinary approach to assessment and treatment is warranted. Management of the most common chronic symptoms falls within the scope of practice of physical therapy, occupational therapy, psychology, and nursing. Social services addressing the financial and functional impact of PCS will also be important.

## Conclusion

The results from the QAPC study provide good information as to the range and severity of problems experienced by people with PCS including patient-centered priorities for intervention in the Canadian context. People with PCS need access to clinical care for management of complications, rehabilitation, and support for self-management of symptoms as appropriate. Further work is needed to identify the most effective interventions and to identify risk and resilience factors in the evolution of symptoms over time.

## Data Availability

Data are available on McGill Dataverse https://www.mcgill.ca/library/services/data-services/sharing/dataverse.
